# Stroke-associated dysarthria

**DOI:** 10.3389/fneur.2025.1629640

**Published:** 2025-08-13

**Authors:** Lan-Xin Lin, Shun-Yu Yao, Quan Chen, Miao-Qiao Du, Xu-Hui Kang, Dai-Yi Jiang, Yong Peng

**Affiliations:** ^1^Department of Neurology, Affiliated First Hospital of Hunan Traditional Chinese Medical College, Zhuzhou, China; ^2^Department of Neurology, Affiliated Provincial Hospital of Hunan University of Chinese Medicine, Zhuzhou, China

**Keywords:** stroke, dysarthria, upper motor neurons, the pyramidal tracts, diagnosis, therapy

## Abstract

A stroke can damage various regions of the brain. Damage to the upper motor neurons of the pyramidal tracts causes unilateral upper motor neuron dysarthria or spastic dysarthria. Dysarthria caused by a stroke is characterized by the coexistence of neurological deficits such as hemiparesis, hemiataxia, clumsiness of one hand, central facial paresis, and tongue deviation. In this review, we summarize the association between stroke and dysarthria, highlight the relevant methods used to measure stroke-associated dysarthria, and discuss specific exercises, advice, explanations, strategies, and psychological support.

## 1 Introduction

Dysarthria is a motor speech disorder caused by damage to the neurological system, resulting in impaired or uncoordinated movement of muscles, including the lips, tongue, lower jaw, velum, vocal cords, and diaphragm. Stroke, traumatic brain injury, and cranial nerve paralysis are the most common neurological disorders causing dysarthria (1). Certain neurodegenerative diseases such as amyotrophic lateral sclerosis (ALS) can also manifest as dysarthria (2). Compared to healthy speakers, in individuals with dysarthria, the vocal articulation space is reduced and concentrated in vocal communication and is characterized by a limited range of motion in the tongue, with more pronounced limitations in the lower jaw and lower lip, slower articulation, and uncoordinated gestures (3, 4).

A stroke can damage different regions of the brain. Damage to the upper motor neurons of the pyramidal tracts causes unilateral upper motor neuron dysarthria or spastic dysarthria (3, 5–7).

In a survey of speech-language therapy provision for individuals with post-stroke dysarthria (PSD) in the UK, approximately half of the patients had dysarthria during the acute phase of stroke (8), after which the prevalence of residual impairment decreased to 27% in the following 6 months (9). Dysarthria after stroke has largely been neglected despite being profoundly disabling (10, 11).

Clinically, dysarthria can be categorized into seven subtypes: ataxic, flaccid, spastic, hypokinetic, hyperkinetic, unilateral upper motor neuron, and mixed. Dysarthria can be caused by various diseases, such as stroke, Parkinson’s disease, cerebral palsy, brain trauma, amyotrophic lateral sclerosis, and brain tumors. Among these underlying disorders, stroke is one of the leading causes of death and adult disabilities, and post-stroke dysarthria (PSD) accounts for more than 20% of all dysarthria cases (3). Dysarthria caused by stroke is characterized by the coexistence of neurological deficits such as hemiparesis, hemiataxia, clumsiness of one hand, central facial paresis, and tongue deviation (1, 12).

Post-stroke spastic dysarthria (PSSD) is an acquired speech disorder that arises from neurological injury and impairs speech intelligibility owing to tense, imprecise, slow, or uncoordinated muscle control. It results in abnormalities in breathing, vocalization, resonance, and rhythm, manifesting as slow and laborious speech, unclear pronunciation, and hypernasality, thereby affecting linguistic function, social participation, and psychological and emotional dimensions (13).

## 2 The association between dysarthria and stroke

Recent neuroimaging studies have shown that PSD is related to lesions in speech-related areas, including the primary motor cortex, lateral premotor cortex, prefrontal cortices, supplementary motor area, corona radiata, internal capsule, striatocapsular area, midbrain, pons, medulla, and cerebellum (14–16). A systematic review of 24 observational studies suggested that brain lesions associated with PSD are located in the supratentorial and infratentorial regions (14).

Dysarthria is a type of communication impairment that commonly occurs after a stroke. It presents in various ways with varying degrees of severity, but it usually leads to reduced intelligibility owing to impaired speech production. Dysarthria affects 52% of stroke survivors. Stroke survivors with dysarthria experience poorer health outcomes, reduced psychological well-being, and more social isolation (17) compared to those with no communication difficulty (4).

Up to one-third of post-stroke survivors experience communication difficulties, including aphasia, dysarthria, or apraxia of speech, which lead to difficulties in language understanding, verbal expression, and writing (18). This synthesis reveals the ongoing difficulties faced by stroke survivors in coming to terms with the loss of communication and adapting to life with communication difficulties (18). Stroke survivors with self-reported communication disabilities appear to have a poorer quality of life between 90 and 180 days post-stroke compared to those without communication disabilities (19). The majority of stroke survivors with communication disabilities experience moderate to extreme levels of anxiety and depression at a greater frequency than those without communication disabilities (19–21). This highlights the need for the early identification of anxiety and depression in individuals with communication problems to facilitate preventative interventions and long-term psychological care (19, 22, 23) (see Table 1).

**Table 1:** Overview of stroke associated dysarthria.

References	Study types	Evaluation index	Results
(1)	Observational study	MRI	Unilateral lingual paresis was detected in all of the patients. The lesions were demonstrated on MRI slices. All lesions were in the same location. The affection of the corticolingual tract without any other motor and sensory tract involvement was proven electrophysiologically.
(13)	Prospective clinical study	FDA, speech articulation, MPT, loudness, and MoCA	At 4 weeks, the experimental group showed significant improvements compared to the control group in the changes in FDA (13.26 ± 6.84 vs. 18.03 ± 5.32, *p* = 0.028), speech articulation (63.17 ± 22.40 vs. 76.51 ± 15.28, *p* = 0.024), MPT (1.34 ± 1.30 vs. 3.89 ± 3.98, *p* < 0.001), loudness (3.46 ± 2.74 vs. 7.14 ± 2.56, *p* = 0.009), MoCA (19.40 ± 3.72 vs. 22.20 ± 5.30, *p* = 0.020), and total effective rate (68.57% vs. 88.57%, *p* = 0.041).
(18)	Systematic review and thematic synthesis	Communication difficulties in relation to day-to-day management	A total of 32 studies were included in the thematic synthesis. The synthesis reveals the ongoing difficulties stroke survivors may experience in coming to terms with the loss of communication and in adapting to life with a communication difficulty. While some were able to adjust, others struggled to maintain their social networks and to participate in activities that were meaningful to them. The challenges experienced by stroke survivors with communication difficulties persisted for many years post-stroke. A total of four themes relating to longer-term needs were developed: managing communication outside of the home, creating a meaningful role, creating or maintaining a support network, and taking control and actively moving forward with life.
(31)	Prospective clinical study	The clinical efficacy, WAB score, GQOLI-74 score, Frenchay dysarthria assessment score, and speech function grades	The overall efficacy in the treatment group was greater than that in the control group (*p* < 0.05). Before treatment, the WAB, Frenchay dysarthria assessment, and GQOLI-74 scores (*p* > 0.05) did not differ between the groups. After therapy, the WAB, Frenchay Dysarthria Assessment, and GQOLI-74 scores in both groups increased significantly (*p* < 0.05), and the treatment group exhibited a significantly greater increase than the control group (*p* < 0.05). Moreover, the classification of speech function did not differ between the two groups before treatment (*p* > 0.05), whereas significant improvements were observed in both groups after treatment (*p* < 0.05). The degree of improvement in the treatment group was greater than that in the control group (*p* < 0.05).
(33)	Systematic review and network meta-analysis	National Institutes of Health Stroke Scale (NIHSS)	Combined acupuncture with language rehabilitation training was the most effective in treating dysarthria symptoms, followed by TA and NA. In addition, the combined effect of acupuncture and language training was superior to that of acupuncture alone. In terms of nerve function recovery, traditional acupuncture and body acupuncture were more effective. To facilitate nerve function recovery, increasing the frequency of acupoints is necessary.
(40)	Prospective clinical study	Auditory perceptual assessment, objective measurement, and National Institutes of Health Stroke Scale (NIHSS)	A total of 67 of 151 participants (44%; mean age = 69 years; SD = 13; 28 female individuals) were diagnosed with dysarthria in the acute phase following stroke. Standardized assessments were possible in 72% (48/67) of the participants. Imprecise articulation of consonants, harsh voice quality, and audible inspiration were the most frequently observed speech characteristics. The acoustic parameters, maximum phonation time and maximum loudness, deviated the most from normative values. UUMN was the main dysarthria type present in 52% (25/48) of the participants. A total of 58% (28/48) and 71% (34/48) of the participants had no, minimal, or mild difficulties at the functional and activity levels, respectively. Speech intelligibility was mildly impaired (median = 91%; IQR = 73–97). According to the NIHSS subitem speech score at hospital admission, 46% (70/151) of the participants had dysarthria, of whom half recovered completely within 1 week after stroke symptom onset.
(48)	Systematic review and meta-analysis of the studies	Acoustic parameters	With our meta-analysis, we analyzed the differences in voice acoustic parameters after speech rehabilitation. The alternating and sequential motion rates (AMR-Pə, AMR-Tə, AMR-Kə, and SMR-PəTəKə) and maximum phonation time significantly improved after speech rehabilitative treatment.
(55)	Single-center randomized controlled trial	Speech breathing level of the modified Frenchay dysarthria assessment	At 3 weeks, there were significant differences between the two groups in the changes in speech breathing level (81% vs. 66%, *p* = 0.011), the modified Frenchay dysarthria assessment (5.54 (4.68–6.40) vs. 3.66 (2.92–4.40), *p* = 0.001), maximum phonation time (5.55 (4.92–6.18) vs. 3.01(2.31–3.71), *p* < 0.01), maximal counting ability (3.08(2.45–3.71) vs. 2.10 (1.53–2.67), *p* = 0.018), and /s/ (3.08 (2.39–3.78) vs. 1.87 (1.23–2.51), *p* = 0.004), while no significant differences were found in the changes in /z/ (3.08 (2.31–3.86) vs. 2.10 (1.5–2.64), *p* = 0.08), s/z ratio (1.26 (0.96–1.55) vs. 1.03 (0.97–1.09), *p* = 0.714), and loudness level (69% vs. 60%, *p* = 0.562).

UUMN, Unilateral upper motor neuron; TA, tongue acupuncture; NA, nape acupuncture; GQOLI-74, Generic Quality of Life Inventory-74; WAB, Western Aphasia Battery; FDA, The Frenchay Dysarthria Assessment scale; MPT, maximum phonation time; MoCA, Montreal Cognitive Assessment scale.

A synthesis of qualitative research demonstrated that the impact of communication difficulties extends beyond the symptomatic manifestation of the medical impairment and influences social relationships, mood, and activities of daily living. The World Health Organization (WHO) International Classification of Functioning, Disability, and Health recognizes the complex interplay of biological, psychological, and social factors that may influence health. The findings from the current review support this model and suggest that more psychosocial factors should be considered in the rehabilitation of patients with post-stroke communication difficulties (18).

In Urban et al.’s (24) study of 68 consecutive patients with sudden-onset dysarthria due to a single infarction, confirmed by magnetic resonance imaging or computed tomography, dysarthria was associated with a classic lacunar stroke syndrome in 52.9% of the patients. Isolated dysarthria and dysarthria-associated central facial and lingual paresis occurred in 2.9% (*n* = 52) and 10.3% (*n* = 57) of the patients, respectively.

**Figure 1:**
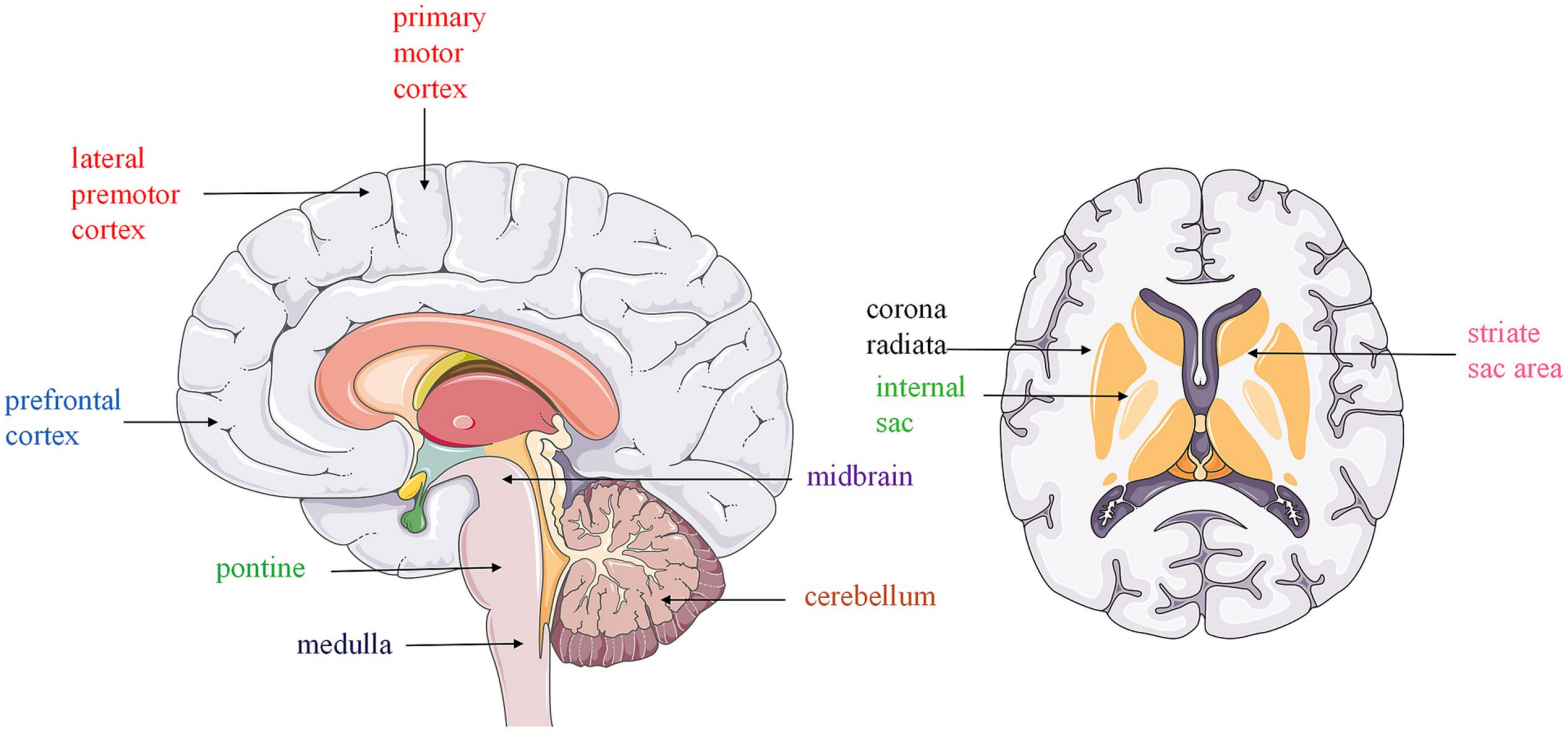
Stroke-associated dysarthria and lesion localization. This figure illustrates the relationship between stroke-associated dysarthria and lesions in key speech-related brain regions. In particular, the primary motor cortex, lateral premotor cortex, prefrontal cortices, supplementary motor area, corona radiata, internal capsule, striatocapsular area, midbrain, pons, medulla, and cerebellum are implicated.

Kayali et al. (1) reported six patients with isolated hypoglossal palsy (IHP) caused by supratentorial ischemic lesions and found that a small lacunar infarction on the corona radiata might cause IHP, and patients with this condition may present with isolated dysarthria. The corona radiata consists of cortical projections in the precentral gyrus of the frontal motor cortex. Following their origin in the frontal motor cortex, the corticobulbar and corticospinal projections pass through the corona radiata. This region receives its vascular supply from the cortical branches of the MCA (25). In this region, the fibers are located too closely to each other. Therefore, ischemic lesions in this region, including lacunar lesions, are generally characterized by several neurological findings with or without lingual paresis (26, 27) (Figure 1).

## 3 Therapies for stroke-associated dysarthria

The “living well with dysarthria” domain highlights the importance of quality of life, psychological well-being, and confidence in communication. Communication impairment has an adverse impact on quality of life (4, 19).

Starting speech therapy immediately after a stroke can enhance recovery. Early, consistent, and intensive treatment results in significantly better outcomes (28). However, despite the recognition of the importance of early intervention, there is a significant lack of clinical studies specifically targeting PSD, particularly in the early stages of stroke (29), underscoring the need for further studies. Furthermore, research is required to determine the benefits and risks of early intervention after stroke (30).

Post-stroke dysarthria belongs to the category of “aphasia” in traditional Chinese medicine. Its pathogenesis includes obstruction of the tongue orifices, a strong tongue, and an inability to speak (31, 32).

Current treatments for stroke-associated dysarthria include breathing exercises, speech training, the Lee Silverman voice treatment, oral motor therapy, proprioceptive neuromuscular facilitation therapy, hyperbaric oxygen therapy, phonation myoelectric stimulation, music therapy, and psychotherapy (33). New therapies, such as stem cell therapy, repetitive transcranial magnetic stimulation (rTMS), transcranial direct current stimulation, motor imagery, virtual reality, acupuncture, Liuzijue Qigong, and novel robotic therapies, have also been introduced in recent years (33, 34).

In dysarthric speech, the vowel space is used for objective acoustic evaluation through the formant measurement of vowels. In particular, the vowel space area (VSA) is associated with the intelligibility of vowels and their correct articulation (35–38). The articulation mechanisms involved in speech disorders with vowel production deficits include reduced excursion and velocity of lingual, lip, and jaw movements and abnormal motion timing (39).

Mou et al. (3) documented the acoustic features of vowel production in Mandarin-speaking patients with PSD; all vowel categories in the patients with PSD showed greater variation than those in healthy speakers. Among the patients, there was a significant overlap between the categories of vowels and reduced vowel space. The magnitude of vowel dispersion and overlap between vowel categories increased as a function of disorder severity. Studying the differences in the public characteristics of patients with post-traumatic disorder and healthy individuals can provide guidance for clinical rehabilitation and improve speech intelligibility in patients with post-traumatic disorder. Articulatory inaccuracy; imprecise consonant articulation; harsh, hoarse, and breathy voice quality; monopitching and loudness; and speech pauses were the most common features observed via auditory–perceptual assessments following a stroke (40, 41). Thus, while assessing patients with dysarthria or swallowing difficulties after stroke, articulation evaluation, including measurements of the vowel space, can be performed to predict dysphagia severity. In addition, speech therapy may be useful in improving swallowing difficulties and preventing complications such as aspiration pneumonia (36).

Ge et al. (42) verified the acoustic characteristics of vowel production in different populations, genders, and tones. The results of this study highlighted the important role played by multiple quantitative acoustic metrics in describing vowel production in Mandarin-speaking clients with PSSD. The second formant (F2) range, VSA, vowel articulation index, and formant centralization ratio are effective in capturing vowel production in PSSD (5, 42).

Over the past few years, various automated speech analysis techniques have been employed to enhance the diagnosis of ALS by detecting subclinical changes. These research efforts have utilized diverse machine learning methods and, in certain instances, attained high levels of diagnostic accuracy through simple tasks, such as sustained phonation of vowels or syllable repetition (2, 43). A clinical investigation was conducted to gather data that could aid in the identification of novel acoustic markers. These markers played a role in the development of a decision tree that effectively stratified the severity of dysarthria, particularly in patients with pronounced speech deficits (43). Based on these speech technologies, dysarthria can be detected, its severity can be assessed, and improvement after stroke treatment can be evaluated.

Interventions for dysarthria typically involve specific exercises, advice, explanations, strategies, or psychological support, depending on the individual’s needs and goals. Impairment-level interventions, such as breathing exercises to improve breath support and control, as well as non-speech oromotor movements to improve the strength, speed, or accuracy of oral muscle movement, may be used. Activity-level strategies to improve intelligibility, such as slowing down speech, overarticulating words, text-to-speech aids, or alphabet charts to spell letters or words, may improve communication success. Psychological support for wider participation may include explanations and education on dysarthria and working with communication partners or communication support groups. Clinical needs often reflect an individual’s previous communication demands and their stage or acceptance of recovery (44). ReaDySpeech is an online program developed through one-on-one interviews and small group discussions with clinicians and patients. As a tailored intervention, it enables therapists to select exercises and activities focused on improving intelligibility. Designed to be user-friendly, accessible, and engaging, it aims to increase uptake and, crucially, treatment intensity (45). Mitchella et al. (45) evaluated the feasibility of a randomized controlled trial on the use of ReaDySpeech for patients with PSD and found that recruitment and retention in this randomized controlled trial of computerized therapy for dysarthria are feasible for patients with acute stroke.

Speech rehabilitation training is commonly used by speech and language therapists as a conventional treatment for PSD (11, 46, 47). Chiaramonte et al. (48) assessed the effectiveness of speech therapy on stroke-related dysarthria: the alternating and sequential motion rate (AMR-Pə, AMR-Tə, AMR-Kə, and SMR-PəTəKə) and maximum phonation time significantly improved after speech rehabilitative treatment.

The rate of speech (ROS) represents the number of words per minute (WPM) spoken by a person. The mean ROS score is used as a diagnostic index and an indicator of success in speech therapy (10). Tamplin showed that ROS levels increased by approximately 10 WPM after music therapy intervention (11).

At present, foreign interventions for dysarthria include a series of strategies such as neurological rehabilitation, behavioral exercise, and social support (49). Behavioral speech exercises can strengthen the breathing and oral muscles to improve speech control. Strategies such as slowing down speech or controlling the pitch are also used to improve speech clarity. Individualized treatment is based on the patient’s prior communication skills, recovery stage, and needs (50).

In Chinese domestic research over the past decade, clinical trials on acupuncture for PSD have shown positive therapeutic effects (51). Su et al. (52) demonstrated that acupuncture, by regulating a suite of molecular signaling pathways involved in redox homeostasis, not only activates the endogenous antioxidant enzyme system but also suppresses the excessive production of reactive oxygen species. Acupuncture intervention can significantly reduce infarct size. In addition, it enhances cerebral blood circulation to promote regional energy metabolism, and it regulates blood lipid metabolism to counteract free radical damage in the brain (53).

Acupuncture can improve nerve sensitivity and promote the recovery of motor function. Tongue acupuncture for the treatment of dysarthria is a new method of acupoint selection that improves the original traditional acupoint selection method by increasing the number of tongue points and manipulation (51).

Tongue acupuncture can increase central nervous system excitability and enhance the functional activity of the language area of the brain (51). A meta-analysis indicated that tongue acupuncture outperformed conventional acupuncture in the management of PSD [OR = 3.62, 95%CI(2.24, 5.85), *p* < 0.0001, I2 = 0.0%] (51) Yang et al. (33) reported that the combination of acupuncture and language rehabilitation training exhibited the most pronounced therapeutic effect in the management of dysarthria symptoms, with tongue acupuncture and nape acupuncture following in efficacy. Furthermore, the synergistic effect of acupuncture combined with language training was superior to that of acupuncture monotherapy.

The acupoints of the tri-tongue needle are located near the tongue root. As the meridians of the liver, heart, Ren, kidney, and spleen all pass through the tongue in different ways, tri-tongue acupuncture (a group of acupuncture points targeting tongue-related disorders) can move local *Qi* and blood, dredge meridians, and regulate viscera (32).

Man et al. (31) adopted low-frequency pulsed electrical stimulation combined with tri-tongue acupuncture to treat PSD. After treatment, the total effective rate in the treatment group was 95.56%, which was significantly higher than that in the control group [82.22% (*p* < 0.05)], suggesting that the combination of low-frequency pulsed electrical stimulation and tri-tongue acupuncture can improve clinical efficacy.

Scalp acupuncture, which is based on traditional acupuncture, is widely used to prevent and treat diseases in specific functional areas of the brain. It has become an effective treatment method for stroke-related dysfunction to reduce negative emotions and improve quality of life in patients with stroke (54).

Liuzijue Qigong, a traditional Chinese fitness exercise compiled by the China Qigong Management Center, is derived from traditional Chinese medicine and involves breathing exercises along with the mantras of the six speech sounds (Xu, He, Hu, Si, Chui, and Xi) (13, 55). Individuals first inhale through the nose and then exhale, forming six distinct tongue and mouth shapes. Simultaneously, they perform proper upper-limb movements to control breathing from top to bottom and vice versa (55, 56). Wang et al. (55) found that Liuzijue Qigong could better improve respiratory control and comprehensive speech ability in patients with stroke-related dysarthria compared to traditional breathing training. Following 3 weeks of intervention, the improvement in speech and breathing function was significantly greater in the Liuzijue Qigong group than in the control group that received basic articulation and traditional breathing training (*p* = 0.011) (55).

Non-invasive brain stimulation (NIBS) methods can monitor and regulate the excitability of intracortical neuronal circuits. Prolonged cortical stimulation may have enduring effects on brain function, thereby justifying the therapeutic use of NIBS in chronic neurological disorders (57). Di Pino et al. (57) proposed that the selection of NIBS should be based on the projected recovery mechanism of each patient. The optimal NIBS protocol depends on the patient’s functional reserve (which determines whether the interhemispheric rivalry model is applicable), stroke type (subcortical vs. cortical or ischemic vs. hemorrhagic), and stroke phase (acute, subacute, or chronic).

Repetitive transcranial magnetic stimulation (rTMS) is a therapeutic technique for post-stroke rehabilitation (58), which generates a magnetic field and induces an electric current that stimulates the superficial brain tissues and depolarizes the neurons of the target cortical tissues (59, 60). It has a neuroprotective effect on the modulation of neuroplasticity, and it improves the brain’s capacity to retrain neural circuits and promotes the restoration and acquisition of new compensatory skills (58). Furthermore, rTMS has been proven to be safe and effective for treating stroke complications. Functional brain activity can be optimized by applying excitatory or inhibitory electromagnetic pulses to the hemisphere ipsilateral or contralateral to the lesion, as well as to the level of the transcallosal pathway, to regulate interhemispheric communication (58).

**Figure 2:**
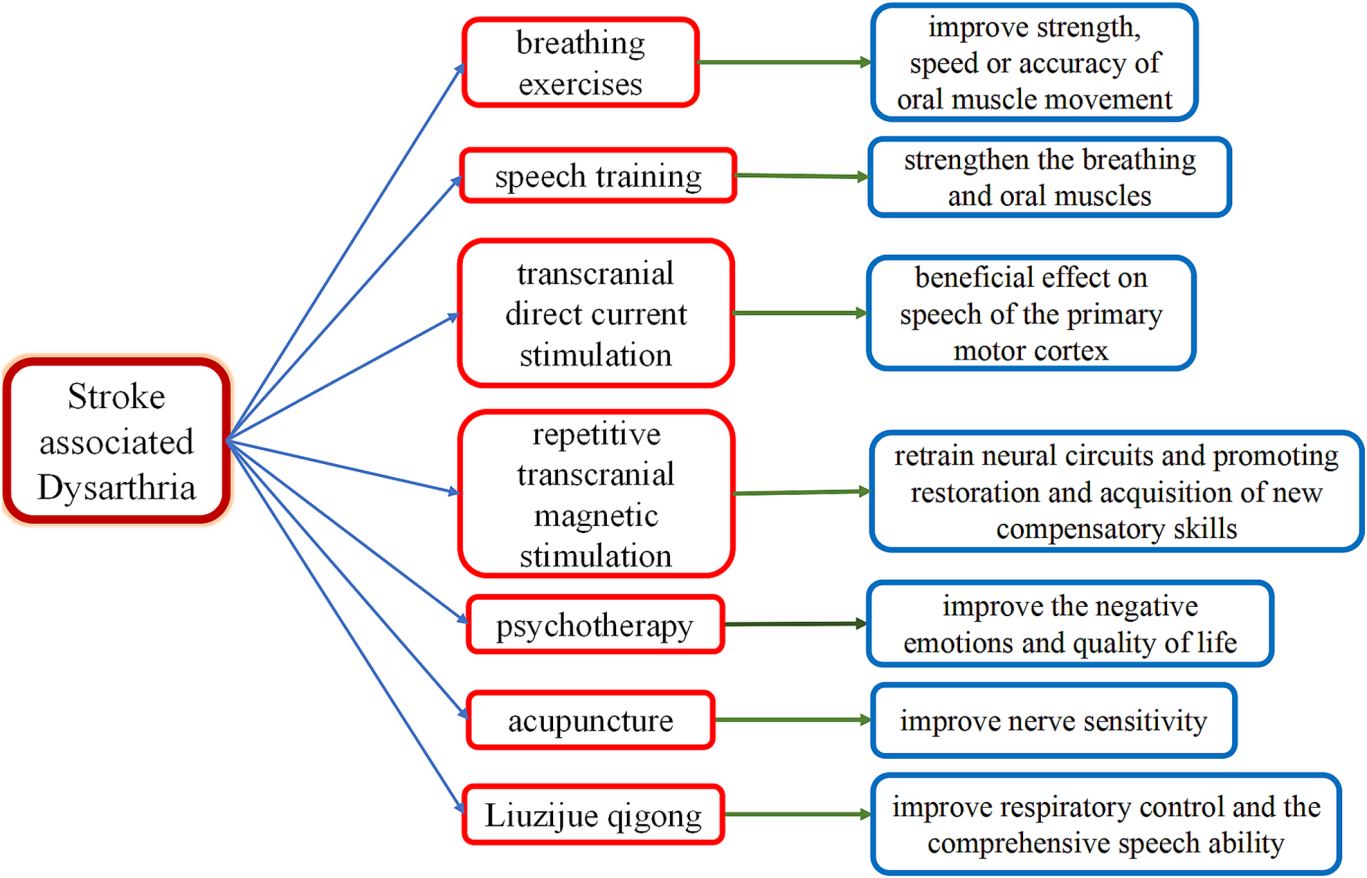
Therapies for stroke-associated dysarthria. This diagram illustrates therapies for stroke-associated dysarthria, including breathing exercises, speech training, psychotherapy, repetitive transcranial magnetic stimulation, transcranial direct current stimulation, acupuncture, and Liuzijue Qigong. Key Mechanisms: breathing exercises: they improve strength, speed, and accuracy of oral muscle movement. Speech training: this strengthens breathing and oral muscles. Psychotherapy: this improves negative emotions and quality of life. Repetitive transcranial magnetic stimulation: This retrains neural circuits and promotes restoration and acquisition of new compensatory skills. Transcranial direct current stimulation: this has a beneficial effect on speech by modulating activity in the primary motor cortex. Acupuncture: this improves nerve sensitivity. Liuzijue Qigong: this improves respiratory control and comprehensive speech ability.

Transcranial direct current stimulation (tDCS) is an invasive neuromodulation method that targets the central nervous system (61). It uses a weak (0.5–2 mA) direct current applied through electrodes on the scalp to shift the resting neuronal membrane potential toward either depolarization or hyperpolarization to change cortical tissue excitability (51). The present study documented the beneficial effect of tDCS on speech in the primary motor cortex, which reversibly polarizes the brain region by applying a mild direct current locally (62, 63). In addition, a combination of tDCS and speech therapy may promote recovery from PSD (62) (Figure 2).

## 4 Discussion

Although the importance of early intervention has been recognized, there is a significant lack of clinical studies specifically targeting PSD, particularly in the early stages of stroke (29).

Currently, speech therapy is cumbersome and repetitive, which negatively affects treatment compliance. In addition, patients may face treatment resource limitations, as speech therapy requires significant time and effort from clinicians. Approximately one-third of patients receive adequate speech therapy. In addition, the amount and frequency of treatment vary across patients (29). Further studies with larger sample sizes are needed to better understand the effects of speech therapy interventions on the mental health of patients with PSD (29).

We need to focus on the ability of patients with stroke-related dysarthria to live well and emphasize the importance of their quality of life, mental health, and communication confidence.
